# Supplementary material for: Mapping the evolution of fertility support policies in China: A content and instrumental analysis
Source: PLoS One. 2025 Oct 9;20(10):e0332137. doi: 10.1371/journal.pone.0332137 (PMC12510515; doi:10.1371/journal.pone.0332137)
Supplement: S1 Appendix — (ZIP) [file pone.0332137.s001.zip › S1 Appendix. 226 original policy documents/120-商务部关于加快城乡便民消费服务中心建设的指导意见(FBM-CLI-4-315461).doc]

商务部关于加快城乡便民消费服务中心建设的指导意见

**发布部门：** [商务部](https://www.pkulaw.com/law/chl?Aggs.IssueDepartment=60220) [机构沿革](javascript:void(0))

**发文字号：**商服贸函〔2018〕157号

**发布日期：**2018.04.19

**实施日期：**2018.04.19

**时效性：** [现行有效](https://www.pkulaw.com/law/chl?Aggs.TimelinessDic=01)

**效力级别：** [部门规范性文件](https://www.pkulaw.com/law/chl?Aggs.EffectivenessDic=XE0302)

**法规类别：** [商贸物资综合规定](https://www.pkulaw.com/law/chl?Aggs.Category=07501) [服务](https://www.pkulaw.com/law/chl?Aggs.Category=07102)

商务部关于加快城乡便民消费服务中心建设的指导意见
（商服贸函〔2018〕157号）

各省、自治区、直辖市、计划单列市及新疆生产建设兵团商务主管部门：
　　为深入贯彻落实党的十九大和中央经济工作会议精神，增强消费对经济发展的基础性作用，推动消费升级，实施乡村振兴战略，促进城乡区域协调发展，满足人民日益增长的美好生活需要，现就加快城乡便民消费服务中心建设工作提出以下意见：
　　**一、**总体要求
　　（一）指导思想。
　　全面贯彻党的十九大精神，以习近平新时代中国特色社会主义思想为指导，紧紧围绕统筹推进“五位一体”总体布局和协调推进“四个全面”战略布局，牢固树立新发展理念，坚持以人民为中心的发展思想，着力推进消费升级和服务消费供给侧结构性改革，构建功能齐备、优质便捷、运营高效、管理有序的城乡便民服务体系，更好满足人民群众日益增长的服务需求，提升人民群众的生活品质，增强人民群众的获得感、幸福感和安全感。
　　（二）基本原则。
　　坚持以人民为中心。以保障和改善民生、增进人民福祉为出发点和落脚点，知民情、了民意、解民忧，着力围绕人民群众最关心最直接最现实的服务需求，构建多层次、全方位、“一站式”的便民服务体系，让城乡便民消费服务中心建设成果更多惠及城乡居民。
　　坚持市场主导和政府引导相结合。充分发挥市场在资源配置中的决定性作用，鼓励社会各方力量积极参与城乡便民消费服务中心建设工作。更好发挥政府作用，针对城乡便民消费服务中心公益性属性和部分居民生活服务业微利薄利特点，加大政策扶持力度，逐步形成政府引导、社会参与、企业投入、市场运作的良性发展机制。
　　坚持保障基本和多元发展相结合。以保障城乡居民基本服务供给为重点，提供安全、便捷、实惠、绿色的大众化服务。支持居民生活服务业集聚发展，加快线上线下融合发展，培育新型、特色、个性化服务模式，满足人民群众多元化的服务需求。
　　坚持横向协作和纵向联动相结合。加强部门间的横向协作，建立协调机制，合力做好城乡便民消费服务中心建设工作。积极争取地方党委政府的重视和支持，将城乡便民消费服务中心建设工作纳入地方的重要民生工程，紧紧依靠城市、街道（乡镇）、社区和大型企业，结合实际，因地制宜，创造性地推动工作。
　　（三）主要目标。
　　从2018年起，力争通过3-5年的时间，推动在中心城市建设一批社区便民服务中心、街区生活服务集聚中心，在农村地区建设一批农村生活综合服务中心；初步建成功能齐备、优质便捷、管理有序、运营高效的城乡便民服务体系，基本满足城乡居民日常生活服务需求，大幅提升城乡居民的生活品质。

　　**二、**主要任务
　　（一）加快建设城乡便民消费服务中心。
　　加快建设社区便民服务中心。结合新型城镇化进程中城市改造、新区建设，鼓励各地政府和有实力的企业，采取政府直接投资、政府和社会资本合作（PPP）、社会资本投资等形式，在中心城市推动新建一批集餐饮、家政、托幼、维修等基本生活服务于一体的便民服务中心。在已建成城市社区，通过公有物业划拨、购买、项目配套、集中连片租赁等方式加强设施建设，通过在现有超市、便利店、菜市场等社区商业设施搭载居民生活服务业等方式完善功能配置，加快改造一批便民服务中心。新建和改造社区便民服务中心具体要求参考《社区便民服务中心建设指南》（附件1）。
　　加快建设街区生活服务集聚中心。结合新型城镇化进程，鼓励各地政府和有实力的企业，采取政府引导和市场化运作相结合的方式，在中心城市推动新建一批各具特色的购物、美食、休闲娱乐和生活服务街区。在已建成城市街区，充分利用现有商业设施，整合服务资源，引导企业合理调整经营品种和结构，加快改造一批各具特色的购物、美食、休闲娱乐和生活服务街区。新建和改造街区生活服务集聚中心具体要求参考《街区生活服务集聚中心建设指南》（附件2）。
　　加快建设农村生活综合服务中心。助力乡村振兴，鼓励各地政府和有实力的企业，采取政府引导和市场化运作相结合的方式，在乡镇推动新建一批乡（镇）生活综合服务中心，作为特色商贸小镇建设的重要内容；在人口聚集的自然村或行政村新建一批村级生活综合服务站。在已建成乡镇，通过公有物业划拨、购买、整合共享等方式加强设施建设，通过在集贸市场、“万村千乡”农家店等商业设施搭载居民生活服务业等方式完善功能配置，加快改造一批乡（镇）生活综合服务中心；在已建成的人口聚集的自然村或行政村，通过集体物业划拨、房产置换、租赁等方式加强设施建设，通过在“万村千乡”农家店等商业设施搭载居民生活服务业等方式完善功能配置，加快改造一批村级生活综合服务站。新建和改造农村生活综合服务中心具体要求参考《农村生活综合服务中心建设指南》（附件3）。
　　各省级商务主管部门可参考上述建设指南，结合各自工作实际，制定本地适用的相关标准，并报送商务部（服贸司）。
　　（二）增强城乡便民消费服务中心服务功能。
　　引导城乡便民消费服务中心不断拓展服务领域、创新服务方式、丰富服务内容。做实基本服务，满足居民的日常生活服务需求；做好选择性服务，满足居民个性化、多元化、多层次的服务需求。城乡便民消费服务中心应以方便城乡居民日常生活为导向，建成以基本服务为主，以选择性服务为补充的多功能的便民服务综合体。
　　（三）激发城乡便民消费服务中心发展活力。
　　鼓励品牌连锁企业通过直营、加盟等形式入驻城乡便民消费服务中心，增强发展活力。鼓励有条件的城乡便民消费服务中心运营公司开展连锁经营，实现规模化发展。充分发挥各类培训机构、技工院校、行业协会及社会团体的作用，加强对城乡便民消费服务中心从业人员的培训，提升服务质量和水平。鼓励农村地区结合实际，引导农民工和大学生等人员返乡参与农村生活综合服务中心建设和运营。
　　（四）创新城乡便民消费服务中心发展模式。
　　鼓励城乡便民消费服务中心及入驻企业，充分借助移动互联、云计算、大数据、人工智能等技术手段，为居民提供多种智慧生活服务，推动数字化、网络化、智能化发展。鼓励城乡便民消费服务中心与大型电商企业合作，整合线上信息资源和线下生活服务资源，推动线上线下融合发展。引导支持城乡便民消费服务中心及入驻企业加快设施设备的节能环保改造，加强废旧物品综合利用，将节能降耗落实到生产经营的各个环节，推动绿色发展。引导城乡便民消费服务中心及入驻企业学习借鉴先进技术和管理模式，从居民需求出发，提高服务精细化、专业化水平，推动品质发展。
　　（五）加强城乡便民消费服务中心运营管理。
　　紧紧依靠基层党组织、政府和群众性自治组织，推动成立由街道（乡镇）、城乡社区、驻社区单位、居民代表、企业等相关各方组成的城乡便民消费服务中心管理机构，引导各方积极参与管理，形成共商、共建、共治、共享的良好氛围和长效机制。及时总结城乡便民消费服务中心运营管理的经验和做法，建立健全运营管理制度，明确政府管理职责、相关各方权利与义务、服务纠纷解决、企业经营需满足的安全环保要求等相关内容。制定科学合理的考核评价体系，开展对本地区城乡便民消费服务中心运营管理情况的督促检查，确保各项运营管理制度落到实处。

　　**三、**保障措施
　　（一）加强组织领导。
　　城乡便民消费服务中心建设事关人民群众最关心最直接最现实的美好生活需要，事关增强消费对经济发展的基础性作用，事关乡村振兴战略的顺利实施。各地商务主管部门要把城乡便民消费服务中心建设作为保障和改善民生的重要任务，增强使命感、责任感和紧迫感，集中力量，真抓实干，明确责任人，制定时间表和路线图，确保各项工作取得实效。
　　（二）合理规划布局。
　　各地商务主管部门要按照新建社区商业和综合服务设施面积占社区总建筑面积比例不低于10%、城乡社区每百户居民拥有综合服务设施面积不低于30平方米等要求，加快推动城乡便民消费服务中心建设专项规划制定工作。强化依规行政的管理理念，研究建立科学的规划实施监督、评估和检查制度。规划发布后，要加强宣传贯彻，提高社会认知度，推动本地城乡便民消费服务中心建设工作加快发展。
　　（三）完善政策措施。
　　各地要着力落实好现有各项政策措施，积极争取小微企业税收优惠、城乡社区综合服务设施供暖、水电、燃气价格优惠等政策，同时要结合各地实际情况，抓紧研究和完善相关配套政策措施，强化政策集聚效应，确保城乡便民消费服务中心建设取得实效。鼓励地方政府采取投资建设、出资购买、公有物业划拨、以奖代补等方式加大城乡便民消费服务中心设施设备投入，在场地租金、贷款贴息等方面给予补贴，或通过购买服务等方式增加居民基本生活服务供给。鼓励各地通过财政资金投入、吸引社会资本参与等方式设立城乡便民消费服务中心发展专项资金，支持入驻城乡便民消费服务中心的品牌、连锁和创新型服务企业做大、做强。
　　（四）鼓励先行先试。
　　鼓励中心城市率先推动建设城乡便民消费服务中心，2018年，推动常住人口1000万以上的城市建设10个以上城乡便民消费服务中心，常住人口500万至1000万的城市建设5个以上城乡便民消费服务中心，其他城市结合需要建设适量的城乡便民消费服务中心。鼓励有条件的农村地区积极探索建设农村生活综合服务中心。各地商务主管部门要主动作为，大胆实践，推动建成一批城乡便民消费服务中心标杆样板项目，形成一批可复制推广的经验和做法。
　　（五）强化监督检查。
　　要制定科学合理的考核评价体系，联合有关部门开展对本地区推进城乡便民消费服务中心建设工作情况的督查评估，摸清建设情况，听取基层干部群众意见建议，挖掘先进典型案例和成功经验，查找突出问题和薄弱环节，不断完善工作举措，促进城乡便民消费服务中心建设工作扎扎实实向前推进。
　　（六）做好业务统计。
　　省级商务主管部门负责统计本地城乡便民消费服务中心建设及运营进度情况，包括已建成、正在建设和计划建设城乡便民消费服务中心数量，已建成城乡便民消费服务中心营业额、服务人次、员工数量等。请各省级商务主管部门每季度统计一次本地城乡便民消费服务中心建设和运营进度情况，填写《城乡便民消费服务中心建设情况统计表》（附件4）和《已建成城乡便民消费服务中心运营情况统计表》（附件5），于下季度初将统计表报送商务部（服贸司）。
　　（七）及时总结推广。
　　各地要充分发挥积极性、主动性和创造性，及时总结可复制、可推广的经验和做法并向商务部（服贸司）报送，通过多种形式进行宣传报道，发挥示范效应，带动全面发展。商务部将深入挖掘各地区和企业的事迹，适时进行宣传报道、经验交流和成果展示，并在更大范围内进行推广。
　　附件：1.社区便民服务中心建设指南
　　2.街区生活服务集聚中心建设指南
　　3.农村生活综合服务中心建设指南
　　4.城乡便民消费服务中心建设情况统计表
　　5.已建成城乡便民消费服务中心运营情况统计表?

商务部
2018年4月19日

　　附件1：

社区便民服务中心建设指南

　　**一、**总体要求
　　（一）本建设指南所指社区便民服务中心是指以社区居民为主要服务对象，以满足社区居民基本生活消费和便民、利民需求为目标，提供公益性和商业性相结合的餐饮、家政、托幼、维修等居民生活服务的便民服务集聚综合体。
　　本建设指南适用于城市社区便民服务中心的新建、改造及经营管理。
　　（二）社区便民服务中心必须符合国家相关法律法规，综合考虑经济社会发展水平，从居民日常生活需求出发，充分利用现有公共服务和商业设施，合理确定建设水平，做到规模适宜、功能完善、安全卫生、运行经济。

　　**二、**建设规模及服务功能要求
　　（一）社区便民服务中心规模应以社区居住人口数量为主要依据，兼顾服务半径确定，营业面积300平方米以上。
　　（二）社区便民服务中心服务功能应与社区的人口结构、居民消费水平、习惯和方式相适应，以满足居民日常生活需求、便捷消费为目标，具体分为基本服务和选择性服务，详见下表。新建社区便民服务中心应优先考虑配置基本服务功能，改造的社区便民服务中心应通过多种途径逐步完善基本服务功能。
　　社区便民服务中心服务功能组合表

| 基本服务 | 选择性服务 |
| --- | --- |
| 大众餐饮（早餐）、家政、理发、维修、生鲜菜店、便利店 | 托幼、老人看护、洗衣、代收代缴、补衣缝纫、康体健身、美容美甲、活动室等 |

　　**三、**选址布局要求
　　（一）社区便民服务中心应选择服务对象相对集中，交通便利，供电、给排水、通讯等市政条件较好，符合消防、食品卫生等安全要求的场所。
　　（二）社区便民服务中心应根据各服务功能的特点，进行合理布局。

　　**四、**经营管理要求
　　（一）社区便民服务中心一般采用统一经营管理的方式，即各项服务的经营权和管理权完全纳入到一个组织机构下，统一规划设计、统一招商经营、统一运营管理，从源头上把控便民服务中心的功能组合、品牌档次，保障项目完整性，最大程度满足社区居民的服务需求。
　　（二）经营场所进行简洁装修，墙壁和地面便于经常清扫刷洗，店内通风、明亮。使用检定合格、未超过检定周期的计量器具。配备消防安全设施或设备，保证消防安全设施齐全、完好、有效。
　　（三）各岗位的从业人员应无传染性疾病，经营食品、副食品的从业人员还应定期进行健康检查，并持有当地卫生主管部门颁发的健康证明。各岗位的从业人员应具有一定的文化程度和与其岗位相适应的业务知识、技术水平，经过社会专门培训机构或企业组织的岗前培训并考试合格。
　　（四）制定职业道德守则，所有岗位从业人员都应遵守，社区便民服务中心定期对从业人员遵守职业道德守则情况进行认真考核。
　　（五）执行国家物价管理政策，所有商品和服务应明码标价。
　　附件2：

街区生活服务集聚中心建设指南

　　**一、**总体要求
　　（一）本建设指南所指街区生活服务集聚中心是指为满足居民购物、美食、休闲娱乐等生活服务需求，统一管理并具有一定规模的生活服务集聚街区，按主要服务功能可分为购物街、美食街、休闲娱乐街和综合生活服务街。
　　本建设指南适用于中心城市街区生活服务集聚中心的新建、改造和经营管理。
　　（二）街区生活服务集聚中心必须符合国家相关法律法规，综合考虑经济社会发展水平，从城市居民日常生活需求出发，充分利用现有公共服务和商业设施，合理确定建设水平，做到规模适宜、功能完善、安全卫生、运行经济。

　　**二、**建设规模及服务功能要求
　　（一）街区的总长度一般应在400米以上，宽度可按街区内设施和人流量确定。
　　（二）街区主要服务功能可以是购物、美食、休闲娱乐等生活服务功能中的一种，也可以是几项生活服务功能的组合。

　　**三、**选址布局要求
　　（一）街区生活服务集聚中心应选择服务对象相对集中，交通便利，供电、给排水、通讯等市政条件较好，符合消防、食品卫生等安全要求的场所。
　　（二）符合所在城市总体规划和商业网点规划的要求，避免盲目冒进、重复建设。

　　**四、**经营管理要求
　　（一）经营场所进行简洁装修，墙壁和地面便于经常清扫刷洗，店内通风、明亮。使用检定合格、未超过检定周期的计量器具。配备消防安全设施或设备，保证消防安全设施齐全、完好、有效。
　　（二）各岗位的从业人员应无传染性疾病，经营食品、副食品的从业人员还应定期进行健康检查，并持有当地卫生主管部门颁发的健康证明。各岗位的从业人员应具有一定的文化程度和与其岗位相适应的业务知识、技术水平，经过社会专门培训机构或企业组织的岗前培训并考试合格。
　　（三）制定职业道德守则，所有岗位从业人员都应遵守，街区生活服务集聚中心定期对从业人员遵守职业道德守则情况进行认真考核。
　　（四）执行国家物价管理政策，所有商品和服务应明码标价。
　　附件3：

农村生活综合服务中心建设指南

　　**一、**总体要求
　　（一）本建设指南所指农村生活综合服务中心是指以农村乡镇、自然村或行政村居民为主要服务对象，以满足居民基本生活消费和便民、利民需求为目标，提供公益性和商业性相结合的大众化居民生活服务的便民服务集聚综合体，按所在位置、规模和服务对象可分为乡（镇）生活综合服务中心和村级生活综合服务站。
　　本建设指南适用于农村生活综合服务中心的新建、改造和经营管理。
　　（二）农村生活综合服务中心项目必须符合国家相关法律法规，综合考虑经济社会发展水平，从农村居民日常生活需求出发，充分利用现有公共服务和商业设施，合理确定建设水平，做到规模适宜、功能完善、安全卫生、运行经济。

　　**二、**建设规模及服务功能要求
　　（一）农村生活综合服务中心项目规模应以乡镇、自然村或行政村居住人口数量为主要依据，兼顾服务半径确定。乡（镇）生活综合服务中心营业面积要求：东中部地区300平方米以上，西部地区200平方米以上。村级生活综合服务站营业面积要求：东中部地区100平方米以上，西部地区80平方米以上。
　　（二）农村生活综合服务中心服务功能应与乡镇、自然村或行政村的人口结构、居民消费水平、习惯和方式相适应，应以满足居民日常生活需求、便捷消费为目标，具体分为基本服务和选择性服务，详见下表。新建农村生活综合服务中心应优先考虑配置基本服务功能，改造的农村生活综合服务中心应通过多种途径逐步完善基本服务功能。
　　农村生活综合服务中心服务功能组合表

| 乡（镇）生活  综合服务中心 | 基本服务 | 选择性服务 |
| --- | --- | --- |
|  | 大众餐饮（早餐）、维修、理发、生鲜菜店、便利店 | 家政、托幼、老人看护、洗衣、代收代缴、补衣缝纫、美容美甲、活动室等 |
| 村级生活  综合服务站 | 基本服务 | 选择性服务 |
|  | 维修、理发、生鲜菜店、便利店 | 大众餐饮（早餐）、家政、托幼、老人看护、洗衣、代收代缴、补衣缝纫、美容美甲、活动室等 |

　　**三、**选址布局要求
　　（一）农村生活综合服务中心应选择服务对象相对集中，交通便利，供电、给排水、通讯等市政条件较好，符合消防、食品卫生等安全要求的场所。
　　（二）农村生活综合服务中心应根据服务功能的特点，进行合理布局。

　　**四、**经营管理要求
　　（一）农村生活综合服务中心一般采用统一经营管理的方式，即各项服务的经营权和管理权完全纳入到一个组织机构下，统一规划设计、统一招商经营、统一运营管理，从源头上把控农村生活综合服务中心的功能组合、品牌档次，保障项目完整性，最大程度满足居民的消费服务需求。
　　（二）经营场所进行简洁装修，墙壁和地面便于经常清扫刷洗，店内通风、明亮。使用检定合格、未超过检定周期的计量器具。配备消防安全设施或设备，保证消防安全设施齐全、完好、有效。
　　（三）各岗位的从业人员应无传染性疾病，具有一定的文化程度，经过岗前培训。
　　（四）执行国家物价管理政策，所有商品和服务应明码标价。
　　附件4：城乡便民消费服务中心建设情况统计表
　　（统计区间：　　　年第　　季度）
　　填报单位：　　　　　　　省（区、市）商务主管部门　　 填报日期：　　 年　 月　 日

| 种类 数量 | 已建成数量  （个） | | 正在建设数量  （个） | | 计划建设数量  （个） | |
| --- | --- | --- | --- | --- | --- | --- |
|  | 新建数量 | 改造数量 | 正在新建数量 | 正在改造数量 | 计划新建数量 | 计划改造数量 |
| 社区便民服务中心 |  |  |  |  |  |  |
| 街区生活服务  集聚中心 |  |  |  |  |  |  |
| 乡（镇）生活  综合服务中心 |  |  |  |  |  |  |
| 村级生活  综合服务站 |  |  |  |  |  |  |
| 合计 |  |  |  |  |  |  |

　　填报说明：
　　1.省级商务主管部门负责填报。
　　2.上表中数量均为统计区间内发生的数量，不是累计数量。其中，“已建成数量”指符合各地相关标准的已建成城乡便民消费服务中心数量。
　　3.省级商务主管部门每季度统计一次本地城乡便民消费服务中心建设情况，并于下季度初将统计表报送商务部（服贸司）。
　　附件5：已建成城乡便民消费服务中心运营情况统计表
　　（统计区间：　　　年第　　季度）
　　填报单位：　　　　　　　省（区、市）商务主管部门　　 填报日期：　　 年　 月　 日

| 便民消费服务中心  种类 | 营业额  （万元，精确到小数点后两位） | 服务人次 | 新增员工人数 |
| --- | --- | --- | --- |
| 社区便民服务中心 |  |  |  |
| 街区生活服务  集聚中心 |  |  |  |
| 乡（镇）生活  综合服务中心 |  |  |  |
| 村级生活  综合服务站 |  |  |  |
| 合计 |  |  |  |

　　填报说明：
　　1.省级商务主管部门负责填报。
　　2.上表中数量均为统计区间内发生的数量，不是累计数量。其中，第一次填报时“新增员工人数”填当时员工人数。
　　3.省级商务主管部门每季度统计一次本地已建成城乡便民消费服务中心运营情况，并于下季度初将统计表报送商务部（服贸司）。

© 北大法宝：（[www.pkulaw.com](https://www.pkulaw.com/)) 专业提供法律信息、法学知识和法律软件领域各类解决方案。北大法宝为您提供丰富的参考资料，正式引用法规条文时请与标准文本核对。
欢迎查看所有[产品和服务](https://www.pkulaw.com/helps/2.html)。法宝快讯：[如何快速找到您需要的检索结果？](https://www.pkulaw.com/helps/70.html)[法宝](https://www.pkulaw.com/helps/69.html)V6有何新特色？

扫描二维码阅读原文

**原文链接：**<https://www.pkulaw.com/chl/b57b774aba7c6062bdfb.html>
